# How to implement geriatric co-management in your hospital? Insights from the G-COACH feasibility study

**DOI:** 10.1186/s12877-022-03051-1

**Published:** 2022-05-02

**Authors:** Bastiaan Van Grootven, Anthony Jeuris, Maren Jonckers, Els Devriendt, Bernadette Dierckx de Casterlé, Christophe Dubois, Katleen Fagard, Marie-Christine Herregods, Miek Hornikx, Bart Meuris, Steffen Rex, Jos Tournoy, Koen Milisen, Johan Flamaing, Mieke Deschodt

**Affiliations:** 1 Department of Public Health and Primary Care, KU Leuven, Leuven, Belgium; 2 Research Foundation – Flanders (FWO), Brussels, Belgium; 3 Jessa Hospital, Hasselt, Belgium; 4 Department of Cardiovascular Medicine, University Hospitals Leuven, Leuven, Belgium; 5 Department of Geriatric Medicine, University Hospitals Leuven, Leuven, Belgium; 6 Department of Cardiovascular Sciences, KU Leuven, Leuven, Belgium; 7 Department of Rehabilitation Sciences, KU Leuven, University Hospitals Leuven, Leuven, Belgium; 8 Department of Anaesthesiology, University Hospitals Leuven, Leuven, Belgium; 9 Competence Center for Nursing, University Hospitals Leuven, Leuven, Belgium; 10 Gerontology and Geriatrics, UZ Herestraat 49, box 7003 35, 3000 Leuven, Belgium

**Keywords:** Implementation, Geriatric, Co-management, Cardiovascular, Geriatric assessment, Nursing, Health services for the aged, Frail, Hospital

## Abstract

**Background:**

Geriatric co-management is advocated to manage frail patients in the hospital, but there is no guidance on how to implement such programmes in practice. This paper reports our experiences with implementing the ‘Geriatric CO-mAnagement for Cardiology patients in the Hospital’ (G-COACH) programme. We investigated if G-COACH was feasible to perform after the initial adoption, investigated how well the implementation strategy was able to achieve the implementation targets, determined how patients experienced receiving G-COACH, and determined how healthcare professionals experienced the implementation of G-COACH.

**Methods:**

A feasibility study of the G-COACH programme was performed using a one-group experimental study design. G-COACH was previously implemented on two cardiac care units. Patients and healthcare professionals participating in the G-COACH programme were recruited for this evaluation. The feasibility of the programme was investigated by observing the reach, fidelity and dose using registrations in the electronic patient record and by interviewing patients. The success of the implementation reaching its targets was evaluated using a survey that was completed by 48 healthcare professionals. The experiences of 111 patients were recorded during structured survey interviews. The experiences of healthcare professionals with the implementation process was recorded during 6 semi-structured interviews and 4 focus groups discussions (*n* = 27).

**Results:**

The programme reached 91% in a sample of 151 patients with a mean age of 84 years. There was a high fidelity for the major components of the programme: documentation of geriatric risks (98%), co-management by specialist geriatrics nurse (95%), early rehabilitation (80%), and early discharge planning (74%), except for co-management by the geriatrician (32%). Both patients and healthcare professionals rated G-COACH as acceptable (95 and 94%) and feasible (96 and 74%). The healthcare professionals experienced staffing, competing roles and tasks of the geriatrics nurse and leadership support as important determinants for implementation.

**Conclusions:**

The implementation strategy resulted in the successful initiation of the G-COACH programme. G-COACH was perceived as acceptable and feasible. Fidelity was influenced by context factors. Further investigation of the sustainability of the programme is needed.

**Trial registration:**

ISRCTN22096382 (21/05/2020).

**Supplementary Information:**

The online version contains supplementary material available at 10.1186/s12877-022-03051-1.

## Background

When hospitalised, older patients frequently experience complications (e.g. delirium) and develop disabilities in their activities of daily living (ADL, e.g. washing or dressing) [1]. This often leads to sustained disability, a lower quality of life and higher costs for society [2, 3]. These observations have voiced the need for a holistic and interdisciplinary approach, i.e., comprehensive geriatric assessment (CGA), when caring for frail older patients in the hospital. CGA refers to “a multidimensional interdisciplinary diagnostic process focused on determining a frail older person’s medical, psychological and functional capability in order to develop a coordinated and integrated plan for treatment and long term follow-up” [4].

Co-management programmes aim to implement CGA-based care on hospital units that have a large proportion of frail patients. They are characterised by shared decision-making and collaboration between a geriatric and non-geriatric team. Although these programmes are often coordinated by a geriatrician, some programmes have an interdisciplinary geriatric team consisting of physicians, nurses and allied health professionals, to prevent and manage geriatric complications. The geriatric team provides care that is complementary to the care typically provided at the specialised wards and support healthcare professionals in the holistic management of frail older patients.

Geriatric co-management has shown clinically beneficial effects on functional status, complications, length of stay and potentially also on in-hospital mortality [5, 6]. Yet, most of the evidence concerns ortho-geriatric units or geriatric fracture centres. These units integrate orthopaedic and geriatric care and focus on preoperative optimisation and management of medical, functional and social needs of older patients with a hip fracture [7]. Frail older patients outside the fracture centers, e.g. on cardiac care wards, are equally at risk for complications, hence they too could benefit from co-management [8, 9]. Outside the geriatric fracture centres, there are no standards available that define the programme components that contribute to successful geriatric co-management [10, 11]. In other words, there is no formal guidance on what interventions to implement in practice, and how to organise a co-management programme to create the desired impact. Finally, none of the studies evaluating the impact of geriatric co-management, have described the strategies that were used to successfully implement geriatric co-management in the hospital.

We therefore developed a nurse-led geriatric co-management programme named G-COACH ‘Geriatric CO-mAnagement for Cardiology patients in the Hospital’ [12] and evaluated the programme in frail patients aged 75 years or older admitted to a cardiac care unit. The findings of our evaluation study (NCT02890927–07/09/2016) demonstrated that the G-COACH programme significantly reduced functional decline, delirium, infections, obstipation and improved quality of life [13, 14].

In this paper, we report the results of the feasibility study that proceeded the evaluation study, as this provides critical insights to clinicians and researchers interested in developing and implementing an in-hospital nurse-led geriatric co-management programme. The specific objectives of the feasibility study were 1) to determine the reach, fidelity and dose of the programme, 2) to evaluate the implementation targets, 3) to describe the experiences of the patients, and 4) to determine implementation determinants for successful initiation and sustainment of the programme.

## Methodology

**Fig. 1:**
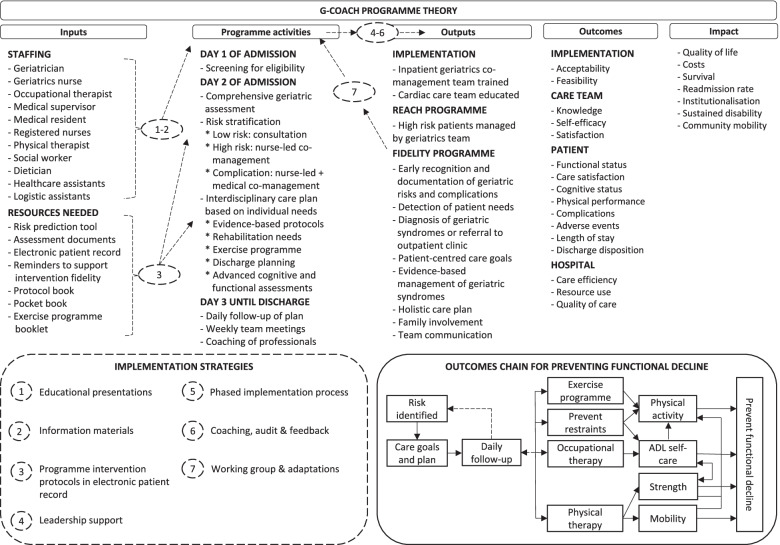
G-COACH Programme Theory. Legend: The figure summarises the programme theory for the G-COACH programme, and defines the inputs necesary to complete the programme activities, which leads to the desired outputs, outcomes and ultimately the impact of the programme. The primary aim of the programme was to prevent functional decline in the hospital so that patients experience less dependency when performing their activities of daily living on the day of hospital discharge. The outcomes chain defines how the programme is expected to achieve this

The G-COACH programme was developed as a new geriatric co-management programme (See Table in Additional file 1 describing the development process) and operationalised for two cardiac care units with 16 and 26 beds, respectively. The programme theory (See Fig. 1) was developed to be applicable for all units, but we chose the cardiac care units as test-units because of the high prevalence of geriatric needs in this population.

### Design

The G-COACH programme was implemented over a 6 month period preceding the feasibility study. The feasibility study was then performed using a one-group experimental design between November 2017 and May 2018. Multiple evaluation methods were used. First, we monitored feasibility indicators to quantify the reach, fidelity and dose. Second, we administered a survey to healthcare professionals to determine if our implementation strategies reached their intended change targets. Third, we conducted a survey to determine how patients experienced and perceived the programme. Fourth, we performed interviews and focus group discussions with healthcare professionals to determine how they experienced the implementation, and how this related to performing the programme. The study was approved by the Medical Ethics Committee of the University Hospitals Leuven (s59543). The study protocol was registered in the ISRCT Registry (ISRCTN22096382–21/05/2020).

### Setting

The study was performed in the University Hospitals Leuven, a 1995 bed teaching hospital, in Belgium. Each cardiac care unit has a multidisciplinary team with a medical supervisor, two medical residents, registered nurses, healthcare assistants, a logistic assistant, a physical therapist, a social worker and a dietician. The inpatient geriatric consultation team has two geriatricians, seven nurses and four occupational therapists that provide geriatric consultations on requests. The geriatrics department aimed to redesign their geriatric consultation service into a geriatric co-management service by changing their team structures and processes.

### G-COACH intervention

When an older patient was admitted to the cardiac care unit, the patient was screened for eligibility in the programme. The following criteria were used: 75 years or older admitted for acute cardiovascular disease or Transcatheter Aortic Valve Implantation and an expected length of stay of at least 3 days. If eligible, the cardiac care nurse submitted an electronic request for the co-management programme. The geriatrics nurse performed a geriatric assessment at the cardiac care unit, ideally within 24 h of admission. The assessment was used to stratify patients in one of three groups (see additional file 3): 1 = patients at low risk for functional decline, 2 = patients at high risk for functional decline, and 3 = patients with acute complications, and to determine the care needs for each patient.

The risk for functional decline was determined using a prognostic model [15], based on the absence/presence of five characteristics, i.e. mobility impairment, cognitive impairment, loss of appetite, depressive symptoms, and use of physical restraints; information that was obtained by the geriatric nurse during the assessment. Mobility impairment was defined as the use of a walking aid before hospital admission as reported by the patient (and equalled 9 points). Cognitive impairment was defined as a Mini-Cog score of less than 3 out of 5 points (and equalled 7 points) [16]. The presence of depressive symptoms was defined as a score > 3 on the 10-item version of the geriatric Depression Scale (and equalled 5 points) [17]. Loss of appetite was defined as self-reported loss of appetite in the past 3 months and was used as a proxy for risk for malnutrition (and equalled 6 points). Use of restraints was defined as the use of physical restraints (e.g., vests, limb ties or chairs with restraints) or an indwelling urinary catheter between admission to the unit and assessment of the predictors (and equalled 5 points). If patients scored in total more than 10 points, which was equivalent to having two or more risk factors present, they were considered at high risk for functional decline.

The presence of acute complications was determined based on a diagnostic assessment of delirium, behaviour problems, urinary retention, urinary incontinence, and malnutrition. These problems were selected in discussion with the geriatrics team and chosen because the team felt that they could impact these outcomes. If a complication was present, the patient was recruited in this group regardless of risk for functional decline.

Patients at low risk for functional decline and without complications received care by the cardiac care team with no further follow-up by the geriatric co-management team, i.e. co-management was not deemed necessary. If indicated, the geriatric team could provide a proactive consult to the cardiac team based on the care needs identified in the geriatric assessment. Patients at high risk for functional decline received daily follow-up by the geriatrics nurse, who developed an individual care plan for the patient with the cardiac care team. Individual care goals were determined with a particular focus on early rehabilitation and discharge planning. The geriatrics nurse was responsible for the coordination of the care plan and supported the cardiac care team with the implementation of protocols for the management of geriatric syndromes. All patients received physical therapy and were motivated to perform an individual exercise programme three times daily without supervision. Patients with cognitive or functional impairments were further assessed by the occupational therapist. If necessary, consultation by a dietician or speech therapist was requested. The patients were discussed with all care professionals in a weekly team meeting at the cardiac care unit. Patients with acute complication received similar follow-up as patients in the group ‘high risk for functional decline’, with additional follow-up by a geriatrician. The geriatrician reviewed the patient file, performed a medication review and prescribed diagnostic investigations when appropriate. A follow-up plan for the complication was discussed first with the nurse from the geriatrics team, who then discussed the plan with the cardiac care team (mostly the nurses and medical residents). The progression of the complication was reviewed on a daily basis. The geriatrician visited the patient bedside when needed, but the nurse was mainly responsible for the bedside follow-up and discussing the plan with the cardiac care team. (See Additional file 3 for a description of the intervention using the TIDieR guidelines).

### Implementation strategies

The implementation of the G-COACH intervention followed the ‘process of change model’ by Grol and Wensing [18], which defines five phases of change: orientation, insight, acceptance, change and maintenance [18]. The maintenance phase was not investigated because of the short study period.

For *orientation*, the goal was to create awareness and cultivate interest and involvement in the G-COACH programme. A stakeholder analysis identified all individuals who were interested in participating in the project. The results of the context analysis were shared with the stakeholders to create a sense of urgency for change. This was supported by promoting the new programme through an email by the head of the department and head nurses, and the publication of an information sheet on the participating units. The G-COACH programme was then formally introduced by the researchers on the participating units with support of the head nurses, explaining the goals and the expected timeline of the project.

For *insight*, the goal was that stakeholders understood what their current performance was, what the G-COACH programme was trying to achieve, and what was expected from them. Educational sessions were organised to inform stakeholders on the prevalence and incidence of geriatric syndromes on their unit, the intended change targets, and how the programme would achieve these by explaining the programme components and protocols.

For *acceptance*, the goal was that stakeholders perceived the programme as valuable and believed it was feasible to perform. Local leaders and champions on the units were asked to promote the programme on the unit and arrangements were made for staffing the geriatric co-management team. The developed protocols were made available and training sessions were organised using case discussions.

For *change*, the goal was to introduce the programme on a small scale so that stakeholders could experience the change and perceive it as a benefit and added value. An official launch of the program was communicated by the head of the department. To facilitate the change, electronic reminders and protocols were programmed in the electronic patient record. The implementation focused on learning the programme. We started with only one patient, discussed with the healthcare professionals how they perceived their competence, and gradually increased the caseload. At the start, researchers monitored the performance bedside allowing for direct feedback and discussion. In a later phase, cases were reviewed using the electronic patient file and individual feedback was given. In the final phase, indicators were monitored using the electronic patient record and feedback was given at the group level to the team of healthcare professionals. Throughout this process, feedback was gathered by the research team and the protocols were further optimised in order to increase acceptability and feasibility. A working group of participating healthcare professionals was allowed to make adjustments to the protocol.

### Sample

In this section, we describe the criteria that were used to recruit patients in the feasibility evaluation. Note that these criteria are slightly different than the criteria used for eligibility in the clinical programme. We believe that all patients who could benefit from co-management should receive it, even if they were not appropriate participants for a feasibility study, e.g. if a research assessment was not possible or if the patient was unable to complete an informed consent. All patients aged 75 years or older were screened within the first 3 days of admission to the participating units. Patients were eligible to participate in the feasibility study if they were admitted for acute cardiovascular disease or Transcatheter Aortic Valve Implantation, had an expected length of stay of 3 days or longer and were not admitted from another hospital or unit (because we did not have baseline data for these patients). Written informed consent was obtained by the researchers. Healthcare professionals were recruited for the evaluation if they had four or more weeks of ‘full time’ experience with the programme. Oral informed consent from the healthcare professionals was obtained by the researchers.

The sample size was informed by the work of Billingham et al. (2013) on the sample sizes of pilot studies [19]. We aimed to recruit 30 patients and 30 healthcare professionals who were exposed to the programme, because we believed this number was sufficient to inform us about the performance of the programme. After the first cohort of 30 patients we performed an interim analysis to identify areas for improvement and gave feedback. This process was repeated two more times. The aggregated data from the three cohorts are reported in this paper.

### Variables

#### Sample characteristics

Demographic data included age, gender and living situation. Baseline clinical characteristics included functional status (Katz Activities of Daily Living Index) [20], cognitive status (Mini Cog) [16], depressive symptoms (Geriatric Depression Scale 10-item version) [17], and nutritional status (Mini Nutritional Assessment short form) [21]. Patient data were collected by researchers on admission to the unit.

#### Feasibility indicators

Feasibility indicators were the reach, fidelity and dose of the programme. The *reach* of the programme was measured using the electronic patient records. A patient was considered to be ‘reached’ if a geriatric assessment and an interdisciplinary care plan was documented in the electronic patient record. The *fidelity* and *dose* of the programme was observed using registrations in the electronic patient record and by patient interviews (e.g. whether or not the patient performed an exercise programme). The fidelity refers to how well the programme was implemented according to the protocol, while the dose refers to how much of the programme was implemented according to the protocol [22]. Indicators were derived from the programme theory and drafted before the start of the study. A standardised checklist was developed to score the indicators and was piloted with the first patients. The researchers monitored the performance on the indicators on a daily basis.

#### Implementation targets

We observed the extent to which our overall programme implementation was successful using an eleven-item survey developed by our research team. The questions were based on the implementation targets that were developed for the implementation strategy: awareness, knowledge, motivation to change, perceived acceptability and feasibility, and believe in the benefit, value and success of the programme. Each question had five response options: completely agree, agree, neutral, do not agree and completely do not agree (see additional file 4 for more details). The survey was piloted internally for ‘readability’ by five nurses and clinician researchers (the intended target population).

#### Patients experiences

Patient experiences were captured using a researcher administered survey. The questions were developed by the research team, internally reviewed, and piloted with the first patients. Nine closed-ended questions informed after the perceived acceptability, usefulness and benefit of the programme. The questions referred to statements about the programme and patients could answer ‘yes’, ‘no’ or ‘neutral’ (see additional file 4) All patients who were recruited in the study were approached by a researcher for the face-to-face survey interview on the day of or the day before discharge from the hospital. The survey was administered in the patients’ room. The response rate was 74% (111/151).

#### Implementation determinants

To capture how healthcare professionals experienced the implementation and determine important determinants (barriers and facilitators) for the implementation, four focus groups and six individual interviews were organised. The participants included nine cardiac care nurses, six geriatrics nurses, two head nurses, a geriatrician, three physical therapists, and six medical residents. One medical resident and social worker declined to participate. One social worker was on leave of absence and could not participate. An interview guide was drafted and was discussed with an experienced qualitative researcher. The interviews were conducted by one researcher in a meeting room at the hospital. The focus groups were conducted by two researchers in a meeting room at the hospital. One researcher was the moderator and the second researcher observed the discussion and took notes. All interviews were tape recorded and written out verbatim. After each interview, a methodological report was drafted, i.e. evaluating the quality of the interview, describing the conditions of the interview, first impressions of important themes, and reflections about how the researcher interpreted the interviews.

### Analysis

The feasibility indicators were reported as frequencies and proportions. Sample characteristics were reported as frequencies and proportions for categorical data and mean and standard deviations for continuous data. The survey data was categorical and reported as frequencies and proportions. A thematic analysis was used to identify the determinants, i.e. reading the transcripts, initial coding, collating codes in themes, reviewing the themes and defining the themes [23]. The process was performed by two researchers and the results were discussed within the research team. Qualitative data were reported using a narrative and thick description.

## Results

### Sample characteristics

**Fig. 2:**
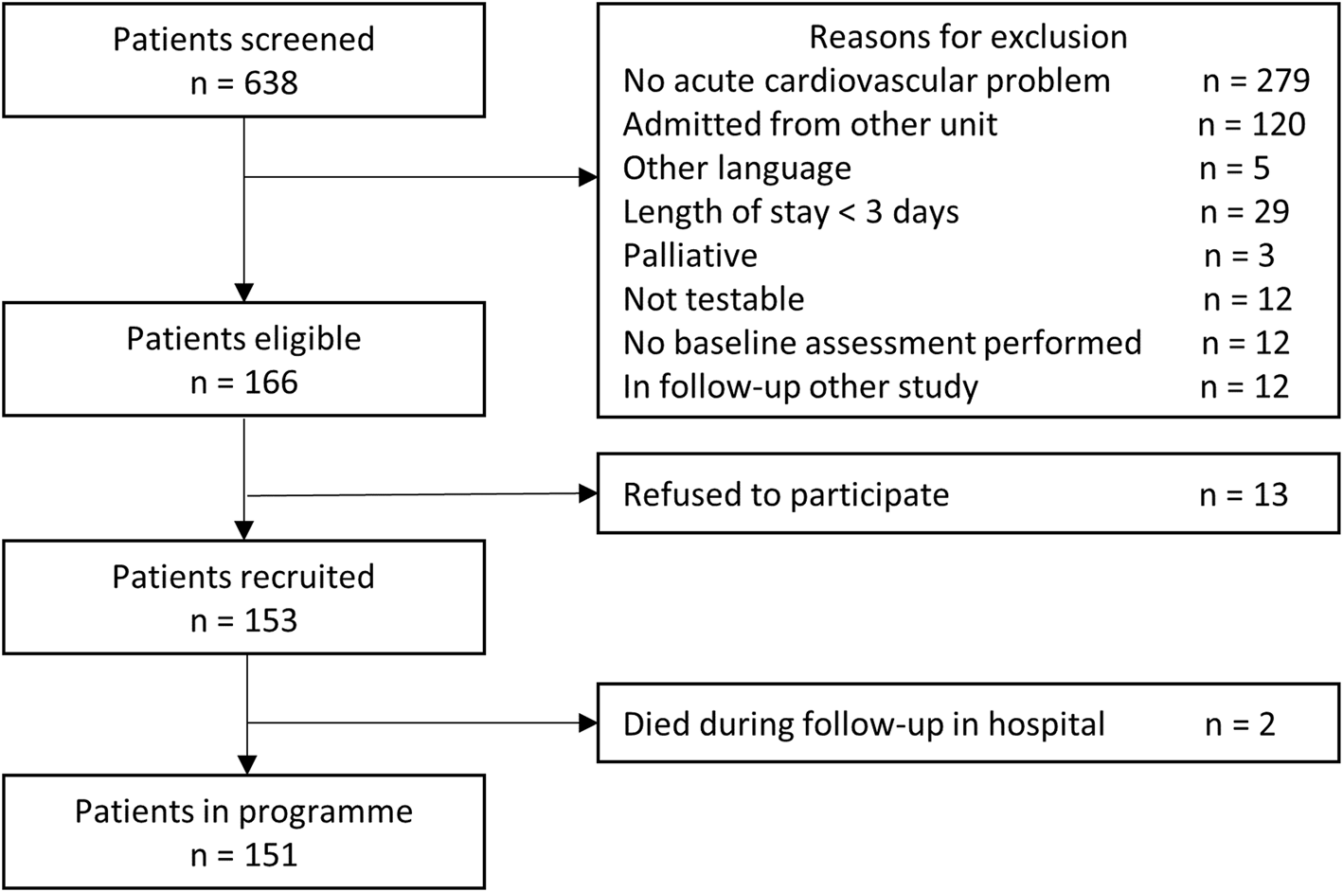
Flowchart of recruitment

A total of 638 patients were screened for inclusion and 151 patients gave their informed consent (see Fig. 2). A total of 52 patients were stratified in the low risk group, 64 in the high risk group, and 35 in the acute complications group.

The mean age was 84 years with men and women being equally represented (see Table 1). On average, patients had two ADL impairments (mean Katz score = 8 points), had moderate to poor cognitive status (mean Mini-Cog score = 2.7) and were at risk for malnutrition (mean Mini Nutritional Assessment score = 10.2).

**Table 1:** Sample characteristics

Characteristics	Sample
Age, mean (SD)	83.8 (4.7)
Male gender, n (%)	76 (50.3)
Living situation, n (%)	
Home	139 (92.1)
Service flat	4 (2.6)
Retirement home	8 (5.3)
Katz ADL index (score 6–18), mean (SD)	8.0 (2.6)
Mini Cog < 3 (score 0–5), n (%)	74 (49.0)
Geriatric Depression Scale (score 0–10), mean (SD)	1.6 (2.1)
Mini Nutritional Assessment (score 0–14), mean (SD)	10.2 (2.4)
Stratification to intervention group, n (%)	
Low risk for functional decline	52 (34.4)
High risk for functional decline	64 (42.8)
Acute complication	35 (23.2)

*Abbreviations*: *ADL* Activities of Daily Living, *SD* Standard Deviation; Note: The values underlined in the scales indicate the ‘best’ score

### Feasibility indicators

The programme reached 91% of the patients, and in 37% of the patients the threshold of starting the programme within 24 h was reached (see Table 2). For 67% of the patients, the programme started within 48 h. The large majority of patients were correctly stratified in the low or high risk for functional decline group, but not in the acute complications group. For 17% of the patients, the wrong stratification resulted in having no care plan and follow-up by the geriatrics team. In patients correctly enrolled in the programme, 98% were co-managed by the geriatrics nurse and the cardiac care team, 83% received rehabilitation by the physical therapist, 69% received discharge planning by a social worker, and 35% completed the individual exercise programme in accordance with the prescribed procedures. Patients who did not complete the prescribed individual exercise programme either did not receive physical therapy (*n* = 8), refused to participate (*n* = 18), received physical therapy but the exercise programme was not instructed (*n* = 7), or were too close to discharge so that starting the programme was not deemed relevant (*n* = 3). In patients correctly stratified in the group with acute complications, 86% received follow-up by a geriatrician and 71% had their medication reviewed.

**Table 2:** Feasibility indicators of the G-COACH programme

Indicators for management by inpatient geriatric co-management team	Adherence
Reach, n (%)	137/151 (91%)
Correct stratification to intervention group, n (%)	
Low risk for functional decline	40/44 (91%)
High risk for functional decline	53/60 (88%)
Acute complication	7/33 (21%)
Patients in programme with follow-up by geriatrics nurse, n (%)	42/43 (98%)^a^
Median number of days to start co-management (IQR)	2 (2)
Start within 24 h of admission, n (%)	16/43 (37%)
Start within 48 h of admission, n (%)	29/43 (67%)
Start within 72 h of admission, n (%)	38/43 (88%)
Median proportion of patients with appropriate follow-up (IQR)	0.50 (0.71)
Patients with documented geriatric risks and complications in electronic patient record, n (%)^b^	43/43 (100%)
Median proportion of geriatric risks accurately documented in electronic patient record (IQR)	0.80 (0.21)
Patients receiving co-management by geriatrician, n (%)	6/7 (86%)
Median proportion of patients with appropriate follow-up (IQR)	1 (0.5)
Median proportion of complications accurately documented in electronic patient record (IQR)	1 (1)
Patients co-managed by geriatrician receiving medication review, n (%)	5/7 (71%)
Documentation of precipitating factors for complications in electronic patient record, n (%)	6/7 (86%)
**Indicators for management of geriatric risks and complications**^c^	
Patients at risk for functional decline receiving physical therapy, n (%)	50/60 (83%)
Patients at risk for functional decline performing an individual exercise program, n (%)	20/58 (35%)^d^
Patients with functional impairments receiving ADL training by an occupational therapist, n (%)	24/39 (62%)
Patients with mobility impairments have access to an ambulatory device on the unit, n (%)	25/29 (86%)
Patients at risk for malnutrition receiving nutritional therapy, n (%)	43/52 (83%)
Median proportion of accurate documentation of nutritional intake during meals (IQR)	0.73 (0.26)
Patients with potential discharge problems receiving discharge planning, n (%)	27/39 (69%)
Patients with potential cognitive impairment receiving cognitive assessment, n (%)	24/36 (67%)
Median proportion of DOSS observations in patients at risk for delirium (IQR)	0.56 (1)
Median proportion of DOSS observations in patients with delirium (IQR)	0.39 (0.58)
Appropriate use of oral laxative or enema for (risk of) obstipation, n (%)	5/6 (83%)
Patients remaining free from a urinary catheter if no indication is present, n (%)	54/60 (93%)
Median proportion of appropriate use of pain medication (IQR)	1 (0.42)
Median proportion of appropriate re-evaluation of pain within 1 h (IQR)	1 (0.81)

*Abbreviations*: *SD* Standard deviation, *IQR* Interquartile range, *DOSS* Delirium Observation Screening Scale; ^a^ Numbers are based on patients who were reached by the programme, correctly stratified and had an active risk status that required follow-up by the inpatient geriatrics co-management team (11 patients did not require follow-up and were not included in the analysis); ^b^ Geriatric risks and complications included the presence or risk for functional decline, falls, cognitive decline, delirium, depression, malnutrition, obstipation, incontinence, urinary retention, pressure ulcers, pain, discharge problems, delirium, behavioural problems; ^c^ Indicators were scored for patients at risk for functional decline and for patients with complications; ^d^ Two missing data

### Implementation targets

A total of 48 healthcare professionals completed the survey on implementation targets (see Table 3). The participants included 35 nurses, three physical therapists, one social worker, two healthcare assistants, four occupational therapists, one dietician and two geriatricians. Almost all healthcare professionals indicated that they knew the programme (98%) and its components (96%), and perceived it as an added value to the care for older patients on the cardiac care units (94%). A total of 94% found the programme acceptable and 74% found it feasible to perform. However, only 49% indicated that the programme was fully integrated in their routine practice.

**Table 3:** Success of implementation targets

Perceptions of healthcare professionals about implementation targets	Sample, n (%)
Healthcare professionals are aware that the programme exists	47/48 (98%)
Healthcare professionals have theoretical knowledge about geriatric risks of older patients on cardiac care units	38/47 (81%)
Healthcare professionals know the components of the programme	45/47 (96%)
Healthcare professionals have knowledge about the specific G-COACH programme protocols	35/47 (75%)
Healthcare professionals are motivated to change their care and participate in the programme	43/47 (91%)
Healthcare professionals perceive the programme as acceptable	44/47 (94%)
Healthcare professionals perceive the programme as feasible	35/47 (74%)
Healthcare professionals perceive the programme as an added value	44/47 (94%)
Healthcare professionals believe that the programme achieved its aim to prevent hospitalisation-associated functional decline	34/47 (72%)
Healthcare professionals believe that if there are problems with the programme, these will be addressed	41/47 (87%)
Healthcare professionals believe the programme has been integrated in the daily routine	23/47 (49%)

### Patient experiences

A total of 95% of patients included in the G-COACH programme found the care acceptable and 96% indicated that the programme addressed their care needs (see Table 4). However, only 72% of patients understood why they received the programme and 63% felt involved in their care. A vast majority of the patients who completed the exercise programme found the individual exercises acceptable (97%), feasible and safe to perform (96%), an added value to their care (89%). About half of the patients (49%) were reminded to perform their exercises. Less than half of the patients (43%) believed that it would improve their functional status.

**Table 4:** Experiences with the programme

Patient experiences with the programme	Sample, n (%)
Patients perceive the programme as acceptable	105/111 (95%)
Patients understand why they are included in the programme	80/111 (72%)
Patients perceive the programme as an added value to their care	80/111 (72%)
Patients perceive the geriatric assessment as acceptable	98/111 (88%)
Patients feel involved in the programme	69/110 (63%)
Patients report that all their needs were addressed by the programme	105/109 (96%)

### Implementation determinants

The interviews with healthcare professionals uncovered 12 themes related to determinants for implementation. First the key themes are shortly described. The most important determinant for the implementation of the programme was the staffing of the geriatric co-management team. The healthcare professionals indicated that the anticipated staffing of a dedicated geriatrics nurse was not available. As a result, the geriatrics nurse had to see patients in the co-management programme on the cardiac care units and also provide consultations on other units on the same day. This was a barrier for two reasons. First, they experienced a conflict in roles because they had to work using their old role (providing advice in a consultation role) and their new role (coaching in a co-management role). Second, they felt that they could not complete the programme as intended which negatively impacted the reach and the fidelity of the programme. The competition in roles and tasks created tension and stress, and was an important barrier to fully integrate the programme on the cardiac care units. Adapting the programme to the context and current needs of the teams was an important determinant for dealing with these stressors. However, they also noted concerns that deviations from the key protocols, because of time constraints, would likely decrease the impact of the programme. The head nurses played a key role in motivating their teams in the participation of the programme, and facilitating the communication with the project team. In the next section, the experiences are reported per theme, and are summarised in Table 5. Quotes are available in Table 5, with references in the text below.

**Table 5:** Implementation determinants related to the experiences of healthcare professionals

Implementation determinants	Information about the determinant	Selected citations from interviews and focus groups
Belief in usefulness	The belief that the programme would be useful to improve the services of the geriatrics department and lead to better patient outcomes fuelled the implementation.	Quote 1: “The literature demonstrated that co-management had better outcomes than geriatric consultation, and we were looking for ways to improve our liaison services. So we wanted to investigate if this model, that focusses more on an integrated collaboration between teams, has better outcomes.” - HP1
Project communication	Personal contacts between the project team and the participating healthcare professionals and informal contacts between the participating healthcare professionals were key in creating awareness of the programme, and were preferred over emails and telephone calls. Information sessions created awareness, but not necessarily knowledge on how to perform the programme. This was likely moderated by the complexity of the change: information was sufficient if the change was simple and small but not if the change was complex or large.	Quote 2: “I know that there was an email about the programme but I probably did not read it. Because if you start your rotation you receive a thousand emails and you have to start planning your care.” - HP27 Quote 3: “The supervisors knew the programme and when you start here on the unit information will find you … I think that somebody from the programme approached me and I had heard of it so I just asked for some more information. That was really sufficient.” - HP23 Quote 4: “The programme was presented several times and we received a lot of information but there is always uncertainty how things will go once we bring the theory to practice.” - HP11
Co-development	A formal needs assessment was necessary for the development of the programme. The assessment had to go beyond quantitative indicators but also include understanding the care culture and routine on the units. The participating healthcare professionals also found it important to understand each other’s needs and care routine. The involvement of local leaders and champions was not sufficient. Involvement needed to reach all participants to facilitate a feeling of ownership. It was key that the programme was not designed as a study but that participants felt that they could tailor it to their needs.	Quote 5: “If nurses from our team work on the project it creates a lot of enthusiasm and we feel that we are part of it.” - HP21 Quote 6: “It is important to reach all nurses. There is a difference between a head nurse that is involved, and all other nurses ... they may not be so motivated. The nurses can’t have the feeling that they have to do it for a study.” - HP1 Quote 7: “You have to understand practically how they are caring for older patients. We need to work together and experience how things are organised on a daily basis. Then it is easier to see how we can improve care for older patients.” - HP4
Scaled implementation	A scaled implementation approach, i.e. start small and build the volume of the programme over time, facilitated the learning process and the implementation. It allowed healthcare professionals to try the program and adjust it, which decreased the resistance to change.	Quote 8: “At the beginning, we were afraid of the workload but once we started it went really well. The change was not drastically and it helped to find areas of the program that were not working well.” - HP12
Learning & skills development	Learning to perform the programme took time and was moderated by several mechanisms: feedback on performance, checklists, protocols and visual reminders. The stakeholders also suggested that case discussions with the entire team would have been helpful. While experimenting with the programme was perceived as useful, complex skills (e.g. coaching) required formal training.	Quote 9: “It is always an adjustment trying something new and it takes time making that transition … You have to experience it and try do to it on your own and learn through the feedback that we received.” - HP8 Quote 10: “The protocols were useful to have. After seeing your patient, you could review what protocols needed to be implemented.” - HP2
Exposure to the programme	A sufficient caseload (exposure/experience) was needed to learn the programme.	Quote 11: “Sometimes it looks as if nurses had not learned from the programme but they may not have had many experience or may not have seen many patients in the programme”. - HP7 Quote 12: “Because of the high staff turnover in medical residents they had not a lot of exposure to the program and were not always well informed.” - HP11
Feedback & adaptations	Adapting the programme to stakeholder feedback was key to ensuring the feasibility. Regular meetings with the project team and a working group with the participating healthcare professionals facilitated this process. The primary concern was workload and staffing levels. The project team had a central role in collecting the feedback and steering the change. This was both a facilitator and a barrier because it may have inhibited the communication between the different teams. It was also noted that adapting the programme too much may limit the clinical impact as key interventions may be compromised.	Quote 13: “It would have been very difficult to perform the original programme with the available staffing levels. The programme has become more feasible but that may also have changed the effectiveness and the expected level of involvement”. - HP8 Quote 14: “With every step, our feedback was asked and there was a lot of willingness to listen and our feedback was always addressed.” - HP18 Quote 15: “At the working group meetings I could easily discuss the programme with my colleagues and what needed to change … because the program is never really finished.” - HP11
Leadership	Head nurses had a key role in motivating the healthcare professionals to change their care routine, address fears for change and perform the programme. The leadership style was an important moderator. However, this also meant that when the head nurse was absent, the performance dropped.	Quote 16: “Once a new head nurse was appointed we knew that additional staff would be hired. That gave us reassurance and we believed that the programme would be more feasible.” - HP4 Quote 17: “I believe that it very much depends on the head nurse, and how they lead the team … you can see it when the head nurse is not present, then things did not go so well.” - HP11
Management support	The perceived lack of support by management to facilitate a good working environment was considered a barrier and was probably related to work motivation.	Quote 18: “Our working environment is not really ideal … and we don’t have the support of the hospital management. If we raise our concerns, nothing happens.” - HP8
Resources	Having dedicated resources for the programme was considered important, which included financial resources for dedicated staffing and having a good work infrastructure.	Quote 19: “The staffing levels will really determine if we can make it a success. Now I know that we really need a dedicated nurse every day for the programme.” - HP8 Quote 20: “Initially you start with project funding so you can experiment. But when the project stops and think its valuable you need to be able to continue it.” - HP1
ICT infrastructure	ICT facilitated the integration of the programme in routine care by becoming more visible. It was perceived that its value was limited by the waiting list by ICT-services.	Quote 21: “If you see the risk score for the patient on your screen, you automatically know that the patient is in the programme and that they are working with the patient.” - HP16 Quote 22: “The Electronic Patient Record has many possibilities but our ICT services need time to programme new modules.” - HP4
Competing tasks	The programme was influenced by the larger strategy of the department. Projects and tasks outside the programme were a barrier to performing the programme.	Quote 23: “Medical residents are not really that involved, they have a lot of other tasks and the project is not high on their priority list.” - HP12 Quote 24: “The geriatrician was supposed to see the patients on the units and discuss the care with the residents, but they have received a lot of new tasks since the start of the programme so they don’t have the time anymore.” - HP9

*Abrreviations*: *HP* Healthcare professional

#### Belief in usefulness

This theme refers to how the belief that the programme would be useful to improve the services of the geriatrics department and lead to better patient outcomes fuelled the implementation. The geriatrics team has historically worked as a consultation service. They reported that they had hoped that their consultations would have had more impact, e.g. prevent complications in older persons. They also had learned that a recent systematic reviews suggested that co-management would be superior to consultation. These insights from the literature made them believe in the potential value of implementing a new programme and facilitated the implementation decision (see quote 1).

#### Project communication

Participants agreed that project communication was an important factor in the implementation. This theme refers to how personal contacts between the project team and the participating healthcare professionals and informal contacts between the participating healthcare professionals were key in creating awareness of the programme (see quote 4), and were preferred over emails and telephone calls. However, they also observed barriers. In particular, the cardiac care nurses and medical residents explained that not everyone is present in these information sessions and because of staff turnover, this alone is not a good strategy. In their experience this was remedied in several ways, including receiving information from colleagues and personal contacts between project team and new staff (see quote 3). Medical residents also referred to an e-mail they received with information about the programme but they did not find this helpful (see quote 2).

#### Co-development

This theme refers to how healthcare professionals were involved in the development of the programme and how the programme was tailored to their needs. Feeling involved from the early stages in the project was experienced as important, and was considered a facilitator for establishing ownership in the programme. Involvement was particularly important for the cardiac and geriatrics nurses and physical therapists, but not for the medical residents. If participants felt less involved, this created a sense of unease about the anticipated implementation and how it will impact them (see quote 6). Participants who felt involved experienced this as a sense of control about the anticipated change and what will be expected from them. For example, this could be established by having healthcare professionals help create the protocols used in the programme. However, such level of involvement was not desired by everyone. Nurses reported a positive experience with the research team learning how they worked and assessed the needs for improvement on the unit (see quote 7). This was also experienced as involvement in the project because they felt that the programme was adapted to the needs, routine and organisation of the unit. Nurses also valued if local leaders or ‘champions’ collaborated with the research team in developing and implementing the programme. The cultivated a sense of involvement at the level of the team and resulted in “enthousiasm” within the team of nurses (see quote 5). However, this was not sufficient to also cultivate a sense of involvement at the level of the individual healthcare professional.

#### Scaled implementation

This theme refers to how the programme was implemented in a sequential manner starting on a small scale and increasing the case-load of the programme. Participants experienced a gradual change in their work routine with the introduction of the programme (see quote 8). They reported that there were some practical problems in the beginning but that these were identified and resolved. For example, the programme defined early physical therapy as a key intervention, but the medical resident did not always complete the prescription for the therapy. They experienced that the case-load of patients in the programme increased over time and stated that starting at full capacity from the start would not have been feasible.

#### Exposure to the programme

This theme refers to how a sufficient caseload was needed to learn the programme. Nurses from the geriatrics team discussed the importance of building experience with the programme in relation to the learning effect of the cardiac care team. They observed a challenge that not all cardiac care nurses work full-time and they have little exposure to the programme (see quote 11). Or that some medical residents only stay a short time on the cardiac are units. They believed that this was related to why some adapted their care to the programme but others did not.

#### Learning and skills development

This theme refers to how healthcare professionals learned to deliver the programme and what helped them in this learning process. This was established through several mechanisms. Nurses stated that they found the checklists, protocols and reminders for the delivery of the programme useful at the beginning to help them in their learning process (see quote 10). They also found it helpful to have the ability to experiment with these, e.g. by having a smaller case-load in the beginning of the implementation. Some nurses experienced an overload of information at the start of the implementation. They found it helpful to receive feedback to help them with the adoption (see quote 9). Some nurses indicated that they would have found it helpful to also have case discussions to help them with the adoption. The geriatrics nurses specifically found it challenging to switch roles between their ‘old way’ of working and what was expected from them in their new roles; i.e. switch from giving advice to become a coach for the cardiac care team. They felt that they needed more formal training to master the new role.

#### Feedback and adaptations

This theme refers to how adapting the programme to stakeholder feedback was key to ensuring the feasibility. The nurses from both teams and physical therapists reported a positive experience with how their feedback was integrated in the project (see quote 14). This was important for them to improve the programme or make it more feasible to perform by suggesting adaptations. For the geriatrics team, adaptations had to be made to reduce the staffing levels in the programme or other activities of the team would had to be stopped. Because of this, they experienced the programme as feasible but were unsure if the full potential was achieved or if a larger effect would be possible with better team staffing (see quote 13). Nurses and physical therapists from the cardiac care team referred to the meetings with the research team and felt that these were important to deal with problems that were not anticipated (see quote 15). They felt that the research team listened to and addressed their concerns and that problems were resolved.

#### Leadership

This theme refers to the role of the head nurses and how they facilitated the implementation within their teams. Nurses from both the cardiac care and geriatrics team experienced that their head nurses were important facilitators for the implementation. This was by expressing their support for and belief in the project, motivating nurses to try adopting the programme, addressing fear for change and uncertainties about the feasibility (see quote 16). Differences between leadership styles of head nurses were observed by the participants. They also noted that the fidelity to the programme was influenced by the presence of the head nurse on the unit. They stated that the fidelity was lower when the head nurse was absent (see quote 17).

#### Management support

This theme refers to how management could support the healthcare professionals. The nurses from the geriatrics team experienced a lack of support by management. They felt that their working environment was very stressful and that addressing this with management did not change anything (see quote 18). They felt standing alone without support and expressed that this influenced their working motivation.

#### Resources

This theme refers to how dedicated resources were needed to adopt the programme. Nurses from the geriatrics team explained how they had to work with a dedicated nurse for the programme that was available on a daily basis (see quote 19). However, they also experienced that this was not always possible depending on the number of requests for the geriatrics team (see quote 20). Overall, they believed that co-management without having a daily nurse available was not feasible.

#### ICT infrastructure

This theme refers to how the ICT infrastructure was used to facilitate the implementation of the programme. They refer to how screening tools and assessment instruments were integrated and the patient profile could be visualised in patient records (see quote 21). However, this was also a barrier as they experienced that ICT support was in high demand in the hospital and that there were long waiting times (see quote 22).

#### Competing tasks

This theme refers to how the fidelity to the programme was influenced by other tasks and responsibilities by participating healthcare professionals. Nurses and physical therapists felt that medical residents were less involved with the programme because they had other tasks which they considered to have higher priority (e.g. attend outpatient clinic; see quote 23). Geriatricians had to take up a new responsibility in a network hospital limiting their time to be available for the programme. This meant that the geriatrician could not always visit patients on the ward (see quote 24). Nurses from the geriatrics team felt that their other tasks in other programmes and projects would become a barrier in the long-term, and that they would need to rethink their responsibility.

## Discussion

This study evaluated the feasibility of implementing a geriatric co-management programme for older patients admitted to the hospital, with cardiac care units used to test the programme. Our results indicate that both patients and healthcare professionals perceive co-management as an added value to conventional care on cardiac care units and that the programme was acceptable and feasible to perform. This was confirmed by the indicators that demonstrated a good reach and fidelity for most of the programme’s core components: geriatric assessment, risk stratification, physical rehabilitation and discharge planning.

To the best of our knowledge, the developed care model is the first publication of a nurse-led geriatric co-management programme for acute hospitalisations. This is different from most co-management programmes that are fully dependent on geriatricians, but who are often not available in clinical practice [24]. The need for healthcare professionals trained in geriatric care and effective care models for older patients will continue to grow with the ageing hospital population. We therefore developed the first formal programme theory, with TIDieR description to support the replication, for a nurse-led inpatient geriatric co-management care model. The development of a programme theory was needed because current evidence regarding co-management is limited to outcome evaluations with poor programme descriptions. Besides describing the programme theory in detail, we also described the first standardised implementation strategy for a geriatric co-management programme. The implementation strategy was based on the relevant behaviour change models and theory which is reported elsewhere [12, 18, 25]. Our qualitative results confirm the importance of multiple constructs of the ‘Consolidated Framework for Implementation Research’. Most notably, the availability of resources in the ‘inner setting’, the adaptability and trialability of the ‘intervention characteristics’, and the champions in the ‘process’ were the key determinants for the implementation [26].

We also learned that our implementation strategy failed to address some important barriers. The staffing levels of the geriatric team, and in particular the geriatricians, prevented sufficient follow-up of acute geriatric complications. Our aim was to use a stakeholder-based development process and adaptive design to fit the programme as best as possible to the context. However, our results infer that structural changes to the context would be needed to improve effective follow-up by the inpatient geriatrics co-management team [27]. For example, the programme was never allotted the defined staffing levels because of competing demands from other projects and tasks in the inner setting. This probably explains why half of the participating healthcare professionals indicated that there was no full integration of the programme into their daily routine.

Furthermore, one of the core components, i.e., the individual exercise programme, was only completed by one third of the patients, mainly because patients were not intrinsically motivated. However, adherence to physical activity is a complex challenge. Patients understand that exercise is important but this does not translate in better adherence to exercise protocols [28]. Several studies have observed a very low level of activity of patients in the hospital, which is associated with functional decline [29–31]. Additional strategies, beyond the reminders that we used, are needed to stimulate the intrinsic motivation and support the self-efficacy of the patients. For example, The SPRINT programme observed that supervision by a health professional was the most important facilitator for patients performing their exercise programme on a geriatrics unit [32].

Several considerations should be made when interpreting our results. For the fidelity and dose indicators, we mostly used registrations in the electronic health records. Not all care actions were registered, and registrations could have been made without the interventions being performed. We also did not assess the quality of the interventions. For example, we only observed whether there was physical therapy and not how well the therapy adhered to the protocol for each individual patient. The interviews were performed by two junior researchers with no prior experience in qualitative analysis. However, the interview guides were discussed with an experienced qualitative researcher. We used self-developed questionnaires to describe the experiences of the participants. Validation was omitted because we were not interested in developing a scale. The evaluation of the feasibility was limited to a few months and we did not collect data on the sustainability of the implementation. Lastly, healthcare professionals were involved in the development of the programme, but patients were not actively involved. This may explain the low fidelity to physical activity exercises. Understanding barriers to physical activity, as experienced by patients, may increase the uptake of this intervention component.

## Conclusion

A stakeholder-centred approach resulted in the successful initiation of a geriatric co-management programme that was perceived acceptable and feasible to perform. Staffing, competing roles and tasks of the geriatrics nurse (i.e., balancing consultation and co-management), and leadership support were key determinants for the implementation. Further research on the sustainability of the implementation is needed.

## Supplementary Information

**Additional file 1.** Development of the G-COACH programme – text.

**Additional file 2.** Development of the G-COACH programme – table.

**Additional file 3.** Description of G-COACH programme based on TIDieR – table.

**Additional file 4.** surveys used in studies.

## Data Availability

The datasets used and/or analysed during the current study are available from the corresponding author on reasonable request, with the exception of interview data to protect the privacy.

## Abbreviations

CGA: Comprehensive geriatric assessment
G-COACH: Geriatric co-management for cardiac patients in the hospital
TIDieR: Template for intervention description and replication
