# Supplementary material for: How to implement geriatric co-management in your hospital? Insights from the G-COACH feasibility study
Source: BMC Geriatr. 2022 May 2;22:386. doi: 10.1186/s12877-022-03051-1 (PMC9059346; doi:10.1186/s12877-022-03051-1)
Supplement: Supplementary file 1 — Additional file 1. Development of the G-COACH programme – text. [file 12877_2022_3051_MOESM1_ESM.docx]

**Additional file 1. Development of the G-COACH programme - text**

We used the ‘purposeful program theory’ methodology for the development of the G-COACH programme ^[[1]](#endnote-1)^. Phases 1 to 3 were used to create a theoretical framework, and phases 4 to 6 were used to operationalise the framework for implementation (See Additional file 2).

***Phase 1: Contextual analysis and evidence review***

First, we performed a context analysis to understand the care needs and outcomes of older patients admitted to the hospital, standard of care practices and local care routines on the cardiac care units, and to assess the potential for change. Focused literature searches were performed to identify evidence on the prevalence and incidence of geriatric syndromes in older patients admitted to the hospital and conceptual models explaining why older patients experience negative outcomes. A pilot cohort study with 63 patients who were admitted to the cardiac care units of the University Hospitals Leuven was performed to assess the quality of care by measuring process and outcome indicators^[[2]](#endnote-2)^. A subsequent independent qualitative study was organised with the purpose of performing participatory observations. One nurse researcher worked on the participating units for 4.5 days to observe the care routines, environment and context, and interviewed the nurses to identify barriers when caring for older persons. Data were used to understand the care context and current practice patterns, and to help explain the performance observed in the pilot observational study.

***Phase 2: Defining the scope of the programme***

The scope of the G-COACH programme was defined in close collaboration with the stakeholders, which included healthcare professionals working on the participating units, but also management and support staff affiliated with the units. The aim was to create a shared understanding regarding the goals and main components of the programme. A systematic review and meta-analysis was performed to identify programme descriptions and outcomes of geriatric co-management and to define the main theoretical components of the programme ^[[3]](#endnote-3)^ ^[[4]](#endnote-4)^. Focus groups and meetings with the head nurses, nurses, medical residents, physical therapists, occupational therapists, social workers and dieticians were organised to identify their interests and expectations for improving the quality of care on their units. Using informal consensus procedures, the aim was to create a shared vision for improvement that was perceived as relevant, acceptable and feasible, and to define the main structural components of the programme.

***Phase 3: Defining the outcomes chain***

We defined an outcomes chain that explains how we expect geriatric co-management to achieve the desired improvements for patients, care professionals and hospital. First, an international two round Delphi study was performed to identify structure, process and outcome indicators. Then stakeholder meetings with hospital management and staff were organised to identify outcomes that were relevant for their strategic decision-making. We hypothesised cause-and-effect relationships between intermediary or process indicators on the one hand, and final or outcome indicators on the other hand.

***Phase 4: Defining the outcome indicators***

We defined a set of outcome indicators that can be used to evaluate the effectiveness of the G-COACH programme. Data from the previously described literature searches, Delphi study and stakeholder meetings were used for this purpose.

***Phase 5: Operationalizing the G-COACH programme***

We developed a manual describing the main components, key processes and responsibilities, and detailing the different protocols for the management of older patients in the hospital. To decrease the tension for change, we used as much as possible the available hospital and team protocols. The research team collated all available protocols and compared them against the available evidence-based guidelines. All changes to the protocols were discussed with and approved by the stakeholders.

***Phase 6: Defining the resources***

We defined the resources that were necessary to perform the protocols by monitored the prevalence and incidence of geriatric syndromes that the programme was expected to manage from hospital admission until discharge. Using these data, we plotted the expected weekly caseload for the programme to determine staffing needs.

1. Funnel SC, Rogers PJ. Purposeful Program Theory: Effective Use of Theories of Change and Logic Models. San Fransisco: Jossey-Bass. 2011. [↑](#endnote-ref-1)
2. Jonckers M, Van Grootven B, Willemyns E, Hornikx M, Jeuris A, Dubois C, et al. Hospitalization-associated disability in older adults with valvular heart disease: incidence, risk factors and its association with care processes. Acta cardiol. 2018:1-7. [↑](#endnote-ref-2)
3. Van Grootven B, Flamaing J, Dierckx de Casterle B, Dubois C, Fagard K, Herregods MC, et al. Effectiveness of in-hospital geriatric co-management: a systematic review and meta-analysis. Age Ageing. 2017;46(6):903-910. [↑](#endnote-ref-3)
4. Van Grootven B, McNicoll L, Mendelson DA, Friedman SM, Fagard K, Milisen K, et al. Quality indicators for in-hospital geriatric co-management programmes: a systematic literature review and international Delphi study. BMJ Open. 2018;8(3):e020617. [↑](#endnote-ref-4)
