# Supplementary material for: How to implement geriatric co-management in your hospital? Insights from the G-COACH feasibility study
Source: BMC Geriatr. 2022 May 2;22:386. doi: 10.1186/s12877-022-03051-1 (PMC9059346; doi:10.1186/s12877-022-03051-1)
Supplement: Supplementary file 2 — Additional file 2. Development of the G-COACH programme – table. [file 12877_2022_3051_MOESM2_ESM.docx]

**Additional file 2. Development of the G-COACH programme - table**

| **Methodology** | **Main findings** |
| --- | --- |
| ***Phase 1: Contextual analysis and evidence review*** | |
| Focused searches in PubMed  Screening reference lists | - Outcomes of hospitalisation: functional decline, delirium, falls, incontinence, unplanned readmission, institutionalisation, death (1-7) - Prognostic factors for negative outcomes: older age, functional impairment, cognitive impairment, impaired mobility, sensory deficits, mood disorders (8, 9) - Conceptual models: dysfunctional model, disability model, predisposing and precipitating risk interaction (1, 10, 11) - Effective care programmes: Acute care for Elders (ACE) units, Hospital Elder Life programme, geriatric co-management programmes (12, 13) |
| Prospective cohort study on the cardiac care units of the University Hospitals Leuven (n= 63 patients) (14) | - Half of the patients developed functional impairments - Half of the patients recovered from their new disability - Physical restraints, urinary indwelling catheters without indication, pressure ulcer strategies and a longer length of stay were determinants for negative outcomes - Despite a multidisciplinary structure, significant delays in rehabilitation, discharge planning and nutritional therapy were observed |
| Participant observations by a nurse researcher on the cardiac care units and the inpatient geriatric consultation team.  Observations: context, environment, structures and processes of care.  Pragmatic open interviews: barriers in daily care  Impressions from the observations were noted at the end of the shift | - High turnover of medical supervisors and residents resulting in a lack of continuity of medical care - Little to no communication and coordination of care between disciplines and often no specific care goals were defined - Delays in starting rehabilitation and discharge planning because information on the patients’ functional and cognitive status, and social situation was missing - Initiation of geriatrics consultation team was often late, with no or minimal interaction between teams and limited follow-up of patients - No integrated care plan addressing the geriatric needs of patients |
| ***Phase 2: Defining the scope of the programme*** | |
| A meta-analysis including 12 intervention studies on geriatric co-management (15) | - Less functional decline, complications and a shorter length of stay - Improved discharge disposition with a structured discharge planning protocol - Improved survival when co-management by an interdisciplinary team - Low GRADE evidence identified potential for better outcomes |
| A systematic review including 46 studies described structures, processes and outcomes of geriatric co-management programmes (16) | - Medical review, discharge planning and rehabilitation were key programme components - Daily patient follow-up and weekly interdisciplinary team meetings were reported in most programme descriptions |
| Stakeholder meetings and focus group discussions to examine  goals and vision of stakeholders  Participants: nurse managers, head nurses, nursing teams, physical therapists, occupational therapists, dieticians, social workers, medical residents, medical supervisors, IT department and care programme managers. | Key areas for improvement were:   - Detection of geriatric syndromes - Delayed discharge - Length of stay - Detection of rehabilitation needs - Team communication   The primary outcome was the prevention of functional decline in patients admitted to the cardiac care units. |
| ***Phase 3: Defining the outcomes chain*** | |
| Hypothesising cause-and-effect relationships between intermediary or process indicators and final outcome indicators | See programme theory (Figure 1) |
| ***Phase 4: Defining the outcome indicators*** | |
| Two round international Delphi study with  33 experts (16) to  identify appropriate and feasible structure, process and outcome indicators for geriatric co-management programme | Key programme indicators were:   - Start co-management within 24 hours of admission - Define selection criteria for the programme (high risk patients) - Protocols for the prevention and management of geriatric syndromes: cognition, delirium, functional status, mobility, falls, pain, medication and pressure ulcers - Key outcomes: length of stay and the incidence of complications |
| Stakeholder meetings were organised to identify the main outcomes that were relevant for the hospital. | Key outcomes were:   - Length of stay (in particular of outlier patients) - Costs - Care efficiency: using resources for the right patient   *See protocol paper for full list of indicators (17)* |
| ***Phase 5: Operationalizing the G-COACH programme*** | |
| Programme protocol development based on hospital protocols, NICE guidelines, and programme descriptions from systematic reviews.  Feedback and consensus meetings with stakeholders. | See programme theory (figure 1) and programme description (Additional file 3) |
| ***Phase 6: Defining the resources*** | |
| Stakeholder meetings and observational data | See programme theory (figure 1) and programme description (Additional file 3) |

**REFERENCES**

1. Covinsky KE, Pierluissi E, Johnston CB. Hospitalization-associated disability: "She was probably able to ambulate, but I'm not sure". JAMA. 2011;306(16):1782-1793.

2. Brennan TA, Leape LL, Laird NM, Hebert L, Localio AR, Lawthers AG, et al. Incidence of adverse events and negligence in hospitalized patients: results of the Harvard Medical Practice Study I. 1991. Qual Saf Health Care. 2004;13(2):145-151.

3. Inouye SK, Westendorp RG, Saczynski JS. Delirium in elderly people. Lancet. 2014;383(9920):911-922.

4. Tinetti ME, Kumar C. The patient who falls: "It's always a trade-off". JAMA. 2010;303(3):258-266.

5. Benbassat J, Taragin M. Hospital readmissions as a measure of quality of health care: advantages and limitations. Arch Intern Med. 2000;160(8):1074-1081.

6. Rudberg MA, Sager MA, Zhang J. Risk factors for nursing home use after hospitalization for medical illness. J Gerontol A Biol Sci Med Sci. 1996;51(5):M189-94.

7. Creditor MC. Hazards of hospitalization of the elderly. Ann Intern Med. 1993;118(3):219-223.

8. Tinetti ME, Inouye SK, Gill TM, Doucette JT. Shared risk factors for falls, incontinence, and functional dependence. Unifying the approach to geriatric syndromes. JAMA. 1995;273(17):1348-1353.

9. Inouye SK, Studenski S, Tinetti ME, Kuchel GA. Geriatric syndromes: clinical, research, and policy implications of a core geriatric concept. J Am Geriatr Soc. 2007;55(5):780-791.

10. Landefeld C. Foreword: The Story of ACE. In: Malone MC EP, RM., editor. Acute Care for Elders A Model for Interdisciplinary Care. New York: Springer Science+Buisiness Media; 2014.

11. Inouye SK. Predisposing and precipitating factors for delirium in hospitalized older patients. Dement Geriatr Cogn Disord. 1999;10(5):393-400.

12. Ellis G, Gardner M, Tsiachristas A, Langhorne P, Burke O, Harwood RH, et al. Comprehensive geriatric assessment for older adults admitted to hospital. Cochrane Database Syst Rev. 2017;9:Cd006211.

13. Hshieh TT, Yang T, Gartaganis SL, Yue J, Inouye SK. Hospital Elder Life Program: Systematic Review and Meta-analysis of Effectiveness. Am J Geriatr Psychiatry. 2018;26(10):1015-1033.

14. Jonckers M, Van Grootven B, Willemyns E, Hornikx M, Jeuris A, Dubois C, et al. Hospitalization-associated disability in older adults with valvular heart disease: incidence, risk factors and its association with care processes. Acta cardiol. 2018:1-7.

15. Van Grootven B, Flamaing J, Dierckx de Casterle B, Dubois C, Fagard K, Herregods MC, et al. Effectiveness of in-hospital geriatric co-management: a systematic review and meta-analysis. Age Ageing. 2017;46(6):903-910.

16. Van Grootven B, McNicoll L, Mendelson DA, Friedman SM, Fagard K, Milisen K, et al. Quality indicators for in-hospital geriatric co-management programmes: a systematic literature review and international Delphi study. BMJ Open. 2018;8(3):e020617.

17. Deschodt M, Van Grootven B, Jeuris A, Devriendt E, Dierckx de Casterle B, Dubois C, et al. Geriatric CO-mAnagement for Cardiology patients in the Hospital (G-COACH): study protocol of a prospective before-after effectiveness-implementation study. BMJ Open. 2018;8(10):e023593.
