# Supplementary material for: How to implement geriatric co-management in your hospital? Insights from the G-COACH feasibility study
Source: BMC Geriatr. 2022 May 2;22:386. doi: 10.1186/s12877-022-03051-1 (PMC9059346; doi:10.1186/s12877-022-03051-1)
Supplement: Supplementary file 3 — Additional file 3. Description of G-COACH programme based on TIDieR – table. [file 12877_2022_3051_MOESM3_ESM.docx]

**Additional file 3**. **Description of G-COACH programme based on TIDieR - table**

| **Brief name** | G-COACH: Geriatric CO-management for ACute Hospiltalisation |
| --- | --- |
| **Why** | Older patients admitted to the hospital have a high risk for developing functional decline. This often results in sustained disability and a lower quality of life. The underlying frailty profile of older patients predisposes them to complications. The hospitalisation context is often harmful for older patients, e.g. patients experience bedrest, are in a strange environment, receive harmful medications and are forced in a dependent position.  G-COACH addresses this at two levels. First, assess the geriatric risks of patients. Second, organise acute care through an interdisciplinary care plan that is based on individual care needs of the patients. |
| **How** | The programme introduced an inpatient geriatric co-management team. Team members from the geriatrics department visited the cardiac care units to coordinate and manage the care for older patients. The introduction of this team on these units was considered the intervention. |
| **Who** | The inpatient geriatric co-management team consisted of   - Geriatrician (0.1 FTE), a medical doctor with specialist training in internal medicine and geriatric medicine and extensive experience working on Acute Care for Elders units and geriatrics consultation services. - Geriatrics nurse (0.5 FTE), a registered nurse with a bachelor degree and extensive experience working on Acute Care for Elders units and geriatric consultation services. - Occupational therapist (0.3 FTE) with a bachelor degree and extensive experience working on Acute Care for Elders units and geriatrics consultation services. |
| **What** | The main components of the programme were:   - Geriatric assessment including the living situation, formal and informal care at home, use of aids and assistive devices, patient and family expectations, functional status, fall history, cognitive status, delirium, depression, behavioural problems, nutritional status, substance (ab)use, voiding, pain, sleep, swallowing and use of physical fixation. - A risk stratification for the outcome functional decline was based on a points score from the geriatric assessment, i.e. mobility impairment = 9 points, cognitive impairment = 7 points, poor appetite = 6 points, depressive symptoms = 5 points, and use of physical fixation or indwelling catheter = 5 points ^[[1]](#endnote-1)^ (Van Grootven et al., BMC Ger 2020). However, patients admitted for transcatheter aortic valve implantation were automatically considered as at risk patients because of their underlying frailty profile. A second stratification was made based on the presence of acute complications: delirium, behavioural problems, new incontinence, new urinary retention, and malnutrition. The patients were stratified in one of three groups:   - Patients at low risk for functional decline (< 10 points): no follow-up by the programme. If indicated, the cardiac care team received a proactive consultation by the geriatric team.   - Patients at high risk for functional decline (≥ 10 points): The patient received daily follow-up of the geriatrics nurse.   - Patients with one or more acute geriatric complications: The patient received daily follow-up of the geriatrics nurse and a geriatrician. - An individualised care plan included   - Rehabilitation needs: A physical therapist focussed on functional strength and mobility. An occupational therapists performed a full assessment focussing on activities of daily living (ADL) if patients were functionally impaired. Based on this assessment, specific ADL training was discussed with the cardiac care nurse.   - Individual exercise programme: A physical therapist instructed patients how to perform basic strength and mobility exercises that could be performed without supervision. To support the exercises, patients received a booklet. The patients were motivated by the therapists and nurses to perform the exercises three times a day. A visual reminder was placed in the patients’ room.   - Discharge planning: A social worker organised the discharge planning. An occupational therapist assessed the patients’ ability and safety to return home, if this need was indicated by the geriatric assessment.   - Implementation of protocols for geriatric syndromes, including functional decline, fall risk, cognitive impairment, behavioural problems, delirium, depressive symptoms, (risk for) malnutrition, (risk for) obstipation, acute urinary incontinence, chronic urinary incontinence, acute urinary retention, (risk for) urinary tract infection, (risk for) pressure ulcers, pain, potentially problematic home situation, medication review. - Daily follow-up on the cardiac care unit was provided by the geriatrics nurse. The follow-up focussed on the implementation of the protocols, coaching of the cardiac care team and reassessing the status of the patients. - Daily team meeting within the inpatient geriatric co-management team were available. The geriatrics nurse had autonomy to coordinate the care for patients at high risk for functional decline, but the geriatrician was available if further discussion was needed. - Weekly team meetings were organised on the cardiac care units, which were attended by the geriatrics nurse. All patients were discussed focussing on the medical treatment, level of nursing care, rehabilitation and discharge planning. |
| **Where** | Two cardiac care units of the University Hospitals Leuven, with 16 and 26 beds, respectively. The unit was staffed with a cardiologist, one to two medical residents, 13.2 to 14.2 FTE registered nurses, 0 to 1.6 FTE healthcare assistants, 2.1 to 3.0 FTE to logistic assistants, 0.35 to 0.4 physical therapists, 0.4 to 0.5 FTE social workers and 0.5 FTE dieticians per unit. |
| **When and how much** | The programme was running on weekdays during office hours, and started the day the patient was admitted to the cardiac care unit. The aim was to include the patient within 24 hours of admission and to start the care plan within 48 hours of admission. The patient remained included in the programme until the day of hospital discharge. |
| **Tailoring** | The protocols were standardised but their implementation was tailored to individual care needs of patients. The inpatient geriatric co-management team was allowed to adjust the protocols based on their clinical expertise. For example, an agreement was made that patients admitted for transcatheter aortic valve implantation did not need daily follow-up by the geriatrics nurse if they demonstrated good postoperative recovery. |
| **Modifications** | Several modifications were made during the feasibility evaluation, based on the feedback of the participating healthcare professionals:   - Initially, nurses were asked to remind patients to perform the individual exercise programme. Based on their feedback, i.e. they forget to remind patients, a visual reminder was added in the patients’ room. - An additional screening document was developed to facilitate the risk stratification by the geriatrics nurse. The screening document was added to the assessment documents. - Initially, the research team screened the patients for the programme, but this responsibility was transferred to the cardiac care nurses. - The nurses reported that the discharge planning process was not optimal and not all social needs were captured, which resulted in delayed discharge. An additional questionnaire was developed to facilitate the assessment of a patients’ social situation. |
| **How well** | The fidelity is detailed in Table 2 of the main manuscript. |

1. Van Grootven, B., Jeuris, A., Jonckers, M., Devriendt, E., Dierckx de Casterlé, B., Dubois, C., . . . Deschodt, M. (2020). Predicting hospitalisation-associated functional decline in older patients admitted to a cardiac care unit with cardiovascular disease: a prospective cohort study. *BMC Geriatrics, 20*(1), 112. doi:10.1186/s12877-020-01510-1 [↑](#endnote-ref-1)
