# Supplementary material for: How to implement geriatric co-management in your hospital? Insights from the G-COACH feasibility study
Source: BMC Geriatr. 2022 May 2;22:386. doi: 10.1186/s12877-022-03051-1 (PMC9059346; doi:10.1186/s12877-022-03051-1)
Supplement: Supplementary file 4 — Additional file 4. surveys used in studies. [file 12877_2022_3051_MOESM4_ESM.docx]

**Additional file 4: surveys used in studies**

**Survey administered to patients**

1) Vindt u het aanvaardbaar dat er een verpleegkundige van de dienst geriatrie bij u is langs geweest?

Nee  Ja  Neutraal

Waarom niet:

2) Is het voor u duidelijk waarom er een verpleegkundige van de dienst geriatrie bij u is langs geweest?

Nee  Ja  Neutraal

3) Vindt u het een meerwaarde dat er een verpleegkundige van de dienst geriatrie bij u langs komt?

Nee  Ja  Neutraal

Waarom wel:

Waarom niet:

4) Vindt u het aanvaardbaar dat er een verpleegkundige van de dienst geriatrie u vragen heeft gesteld over uw … ?

Thuissituatie  Nee  Ja  Neutraal

Dagelijks functioneren  Nee  Ja  Neutraal

Geheugen, mentale toestand  Nee  Ja  Neutraal

Voedingsinname  Nee  Ja  Neutraal

Symptomen van depressie  Nee  Ja  Neutraal

5) Kon u tijdens het gesprek met de verpleegkundige van de dienst geriatrie aangeven dat u bepaalde zorgen of vragen heeft over uw … ?

Thuissituatie  Nee  Ja  Neutraal

Revalidatie  Nee  Ja  Neutraal

Opname in het ziekenhuis  Nee  Ja  Neutraal

Ontslag  Nee  Ja  Neutraal

Zorgen na ontslag uit het ziekenhuis  Nee  Ja  Neutraal

6) Was er informatie die voor u belangrijk is maar dat u niet kon vertellen tijdens uw gesprek met de geriatrisch verpleegkundige?

Nee, alle belangrijke zaken werden besproken.

Ja,

Reden:

7) Voelde u zich voldoende betrokken bij uw … ?

Behandelplan  Nee  Ja  Neutraal

Dagelijkse zorg  Nee  Ja  Neutraal

Revalidatie  Nee  Ja  Neutraal

Ontslagplanning  Nee  Ja  Neutraal

8) Zijn er zorgen over uw behandeling, zorg, revaluatie of ontslagplanning die u niet heeft kunnen aangeven of bespreken met hulpverleners van cardiologie en/of geriatrie?

Nee  Ja  Neutraal

Wat niet:

9) Vindt u het een meerwaarde dat u wordt gevolgd door hulpverleners van cardiologie en geriatrie en dat deze hulpverleners samenwerken om u zorg te verlenen?

Nee  Ja  Neutraal

Reden:

| Ik ken de doelstellingen van het G-COACH programma. |  | ○ | ○ | ○ | ○ | ○ |
| --- | --- | --- | --- | --- | --- | --- |
|  |  | volledig akkoord | akkoord | neutraal | niet akkoord | volledig niet akkoord |
| Ik weet wat het G-COACH programma inhoudt. |  | ○ | ○ | ○ | ○ | ○ |
|  |  | volledig akkoord | akkoord | neutraal | niet akkoord | volledig niet akkoord |
| Ik heb voldoende achtergrondkennis over de geriatrische problemen van oudere patiënten op de afdeling Hart- en Vaatziekten (E433, 435). |  | ○ | ○ | ○ | ○ | ○ |
|  |  | volledig akkoord | akkoord | neutraal | niet akkoord | volledig niet akkoord |
| Ik vind het G-COACH programma een meerwaarde in de zorg aan ouderen op de afdeling Hart- en vaatziekten. |  | ○ | ○ | ○ | ○ | ○ |
|  |  | volledig akkoord | akkoord | neutraal | niet akkoord | volledig niet akkoord |
| Ik vind dat het G-COACH programma functionele achteruitgang voorkomt bij patiënten. |  | ○ | ○ | ○ | ○ | ○ |
|  |  | volledig akkoord | akkoord | neutraal | niet akkoord | volledig niet akkoord |
| Het GST betrekt mij bij het opstellen van een geriatrisch zorgplan bij patiënten in het G-COACH programma. |  | ○ | ○ | ○ | ○ | ○ |
|  |  | volledig akkoord | akkoord | neutraal | niet akkoord | volledig niet akkoord |
| Ik voel mij gemotiveerd om samen te werken met het GST voor patiënten in het G-COACH programma. |  | ○ | ○ | ○ | ○ | ○ |
|  |  | volledig akkoord | akkoord | neutraal | niet akkoord | volledig niet akkoord |
| Ik vind het G-COACH programma haalbaar om uit te voeren. |  | ○ | ○ | ○ | ○ | ○ |
|  |  | volledig akkoord | akkoord | neutraal | niet akkoord | volledig niet akkoord |
| Bij problemen met het G-COACH project heb ik er vertrouwen in dat deze worden opgelost. |  | ○ | ○ | ○ | ○ | ○ |
|  |  | volledig akkoord | akkoord | neutraal | niet akkoord | volledig niet akkoord |
| Ik vind het aanvaardbaar dat G-COACH geïmplementeerd is op de afdeling Hart- en Vaatziekten. |  | ○ | ○ | ○ | ○ | ○ |
|  |  | volledig akkoord | akkoord | neutraal | niet akkoord | volledig niet akkoord |
| Ik vind dat het G-COACH programma voldoende ingeburgerd is in de dagelijkse werking van de afdeling Hart-en Vaatziekten. |  | ○ | ○ | ○ | ○ | ○ |
|  |  | volledig akkoord | akkoord | neutraal | niet akkoord | volledig niet akkoord |
| Ik vind dat ik over de nodige kennis en kunde beschik om de zorg aan patiënten in het G-COACH programma uit te voeren. |  | ○ | ○ | ○ | ○ | ○ |
|  |  | volledig akkoord | akkoord | neutraal | niet akkoord | volledig niet akkoord |

**Survey administered to cardiac care team**

**Survey administered to geriatrics team**

| Ik ken de doelstellingen van het G-COACH programma. |  | ○ | ○ | ○ | ○ | ○ |
| --- | --- | --- | --- | --- | --- | --- |
|  |  | volledig akkoord | akkoord | neutraal | niet akkoord | volledig niet akkoord |
| Ik weet wat het G-COACH programma inhoudt. |  | ○ | ○ | ○ | ○ | ○ |
|  |  | volledig akkoord | akkoord | neutraal | niet akkoord | volledig niet akkoord |
| Ik heb voldoende achtergrondkennis over de geriatrische problemen van oudere patiënten op de afdeling Hart- en Vaatziekten (E433, 435). |  | ○ | ○ | ○ | ○ | ○ |
|  |  | volledig akkoord | akkoord | neutraal | niet akkoord | volledig niet akkoord |
| Ik vind het G-COACH programma een meerwaarde in de zorg aan ouderen op de afdeling Hart- en Vaatziekten. |  | ○ | ○ | ○ | ○ | ○ |
|  |  | volledig akkoord | akkoord | neutraal | niet akkoord | volledig niet akkoord |
| Ik vind dat het G-COACH programma functionele achteruitgang voorkomt bij patiënten. |  | ○ | ○ | ○ | ○ | ○ |
|  |  | volledig akkoord | akkoord | neutraal | niet akkoord | volledig niet akkoord |
| Ik vind dat hulpverleners van de afdeling Hart- en Vaatziekten betrokken zijn bij het opstellen van een geriatrisch zorgplan voor patiënten in het G-COACH programma. |  | ○ | ○ | ○ | ○ | ○ |
|  |  | volledig akkoord | akkoord | neutraal | niet akkoord | volledig niet akkoord |
| Ik voel mij gemotiveerd om samen te werken met de hulpverleners van de afdeling Hart- en Vaatziekten voor patiënten in het G-COACH programma. |  | ○ | ○ | ○ | ○ | ○ |
|  |  | volledig akkoord | akkoord | neutraal | niet akkoord | volledig niet akkoord |
| Ik vind het G-COACH programma haalbaar om uit te voeren. |  | ○ | ○ | ○ | ○ | ○ |
|  |  | volledig akkoord | akkoord | neutraal | niet akkoord | volledig niet akkoord |
| Bij problemen met het G-COACH project heb ik er vertrouwen in dat deze worden opgelost. |  | ○ | ○ | ○ | ○ | ○ |
|  |  | volledig akkoord | akkoord | neutraal | niet akkoord | volledig niet akkoord |
| Ik vind het aanvaardbaar dat G-COACH een onderdeel is van de activiteiten binnen het GST. |  | ○ | ○ | ○ | ○ | ○ |
|  |  | volledig akkoord | akkoord | neutraal | niet akkoord | volledig niet akkoord |
| Ik vind dat het G-COACH programma voldoende ingeburgerd is in de dagelijkse werking van de afdeling Hart-en Vaatziekten. |  | ○ | ○ | ○ | ○ | ○ |
|  |  | volledig akkoord | akkoord | neutraal | niet akkoord | volledig niet akkoord |
| Ik vind dat ik over de nodige kennis en kunde beschik om de zorg aan patiënten in het G-COACH programma uit te voeren. |  | ○ | ○ | ○ | ○ | ○ |
|  |  | volledig akkoord | akkoord | neutraal | niet akkoord | volledig niet akkoord |
